# Spending on and Use of Clinician-Administered Drugs in Medicare

**DOI:** 10.1001/jamahealthforum.2023.2941

**Published:** 2023-09-08

**Authors:** Megan F. Hyland, Rebecca M. Sachs, Lara Robillard, Tamara B. Hayford, Ge Bai

**Affiliations:** 1 Jeb E. Brooks School of Public Policy, Cornell University, Ithaca, New York; 2 Health Analysis Division, Congressional Budget Office, Washington, DC; 3 Budget Analysis Division, Congressional Budget Office, Washington, DC; 4 Health Analysis Division, Congressional Budget Office, Washington, DC; 5 Johns Hopkins Carey Business School, Baltimore, Maryland; 6 Johns Hopkins Bloomberg School of Public Health, Baltimore, Maryland

## Abstract

**Importance:**

Medicare Part B drug expenditures have increased in recent years. This trend is likely to persist given the increased use and availability of biologics.

**Objectives:**

To assess the extent to which Medicare Part B spending growth was associated with changes in price vs quantity, and how these trends interacted with entry of new drugs into the marketplace.

**Design, Setting, and Participants:**

This cross-sectional study quantified the degree of spending concentration and the association between price and use of Part B drugs among fee-for-service Medicare beneficiaries. Data on use and spending for separately payable Part B drugs were included. Source data were aggregated to the drug-year level and reported from 2016 to 2020. Descriptive decomposition and index analyses quantified the relative association of price and use changes separately for existing single-source drugs, existing drugs that faced competition, and new drugs that entered the market. Data analysis was performed from June to December 2022.

**Main Outcomes and Measures:**

Part B drug spending by the fee-for-service Medicare program and beneficiaries, as well as use, defined as dosage units and beneficiaries using the drugs.

**Results:**

The study included 535 unique Part B drug products. From 2016 to 2020, 15 or fewer products comprised half of all Part B drug expenditures. The set of 7 drugs that comprised the top 25% of spending was very consistent over time, and all were biologics. Part B drug products that cost $1.85 or less per administration accounted for more than half of the doses administered in 2020. Spending on Part B drugs increased by $15 billion from 2016 to 2020. The entry of new, nonbiosimilar drugs during this period accounted for $12 billion of this increased spending (80%), while shifts in use and price increases among existing single-source brand drugs accounted for the remaining increase in spending. Part B spending decreased among the subset of existing drugs facing generic or biosimilar competition. Among single-source drugs on the market in 2016, the index that varied dosage units exceeded the index that varied price in all years, confirming that changes in use were associated more with spending growth for those drugs.

**Conclusions and Relevance:**

In this cross-sectional study of Medicare Part B drug expenditures, spending was found to be concentrated among a small number of drugs. The entry of new products was a key factor associated with recent increases in Part B drug spending. These findings suggest that policies targeting top-selling drugs may have greater potential to curb Part B drug spending than those targeting price growth.

## Key Points

**Question:**

To what extent are changes in price and use among new and existing drugs associated with recent increases in Medicare Part B drug spending?

**Findings:**

In this cross-sectional study including 535 separately payable Part B drugs, the introduction of new drugs accounted for 80% of Part B spending growth from 2016 to 2020, with much of the remainder stemming from changes in the use of existing single-source drugs.

**Meaning:**

These findings suggest that market entry was associated with much of the growth in Part B drug spending.

## Teaser

This cross-sectional study assesses the extent to which Medicare Part B spending growth was associated with changes in price vs quantity, and how these trends interact with entry of new drugs into the marketplace.

## Introduction

Medicare Part B is the medical insurance benefit that covers drugs administered by infusion or injection in clinician offices and hospital outpatient departments (HOPDs). Common examples include drugs to treat cancer, rheumatoid arthritis, and macular degeneration. Among other clinician and outpatient services, Part B drug benefits also extend to vaccines and certain drugs furnished by pharmacies and suppliers, although these products constitute a minority of all drugs covered.

In 2020, nearly 4 million beneficiaries of traditional Medicare used Part B–covered drugs, for which total payments reached $38.5 billion.^1^ Although total Part B spending was nearly one-fifth of Medicare’s outpatient prescription drug benefit (Part D) in that same year,^1^ Part B spending has increased at a faster rate. From 2008 to 2021, Part B drug spending per fee-for-service (FFS) enrollee increased 9.2% per year compared with the 2.6% increase in Part D spending.^2^ Such sustained increases in spending can put pressure on Medicare program reserves and spur increases in beneficiary premiums. In addition, beneficiaries of traditional Medicare face 20% cost sharing for most Part B drugs. Thus, high prices can also impose significant financial burdens, especially for the 5.6 million traditional Medicare enrollees without supplemental coverage.^3^

Controlling Part B spending is an active area of policy interest. Beginning in 2023, the Inflation Reduction Act of 2022 required manufacturers of some Part B drugs to pay a Medicare rebate when prices increased faster than the rate of inflation.^4^ This provision may curb future increases in drug prices and coinsurance amounts. Select high-spending Part B drugs will also be subject to negotiated drug prices beginning in 2028.

This cross-sectional study aimed to measure the concentration of and increase in Part B drug spending. We assessed the factors associated with the increase in spending by examining the concentration of spending and by decomposing spending increases into changes in price vs quantity for existing single-source drugs, existing drugs facing competition, and new drugs entering the market. We found that spending on Part B drugs was concentrated among a small number of products each year, and the entry of new products was a key factor associated with the increase in spending from 2016 to 2020. This analysis can help policymakers understand how different components of spending are associated with increased growth over time.

## Methods

This cross-sectional study was not submitted for institutional review board approval because it used nonidentifiable public data and did not constitute human participant research (45 CFR §46.102). This study adheres to the Strengthening the Reporting of Observational Studies in Epidemiology (STROBE) reporting guideline for cross-sectional studies.

### Data Source and Analysis Sample

We used Medicare Part B Spending by Drug data on separately payable drugs administered to all FFS beneficiaries in HOPDs and clinician offices from January 2016 to December 2020. These data excluded lower-cost HOPD drugs packaged with other services under a single rate, as well as drugs billed under “not otherwise classified” billing codes. The latter restriction limited our ability to capture periods when a drug was approved for use but not yet assigned a Healthcare Common Procedure Coding System (HCPCS) code. We further excluded products that were not subject to the standard Medicare payment limit, which included radiopharmaceuticals, skin products, and vaccines.

For each drug HCPCS code and year, we observed the Medicare payment limit, total spending, and the number of dosage units and beneficiaries using the drug. The Medicare payment limit reflected the mean price manufacturers realized 2 quarters prior, less rebates, discounts, and price concessions. In general, clinicians received a fixed amount plus a 6% add-on to account for variability in clinician acquisition costs. During our analysis period, sequestration reduced the add-on to 4.3%, although our data listed the full payment amount. Because HCPCS codes are updated and replaced over time, we compiled data across annual releases and used name and dosage information to streamline identifiers. Because our analysis compares changes over time, we excluded drugs that appeared intermittently in the panel. The 38 intermittent drugs accounted for no more than 0.1% of total Part B spending and dosage units in each year; some were discontinued in the US, several had an oral Part D equivalent, and for others, prescribing volumes dipped below the censoring threshold of the Centers for Medicare & Medicaid Services (CMS). Our final data set contained 535 unique Part B drug products (eFigure 1 in Supplement 1).

We supplemented the Medicare Part B Spending by Drug data with CMS’ annual Average Sales Price Drug Pricing Files, which list the national drug codes affiliated with each HCPCS code. We created an indicator for whether a drug ever faced generic competition by counting the number of unique labeler codes for a given drug HCPCS code in the October 2020 file. Drugs with 1 labeler were considered single-source brand drugs, and all others were classified as generic. To inform how many of these drugs faced competition for the first time during our sample period, we also created a generic indicator with the January 2016 file. To better account for competitive effects among biologics in the decomposition and index analyses, we grouped reference biologics with their launched biosimilars. For these combined products, we summed dosage units across all relevant reference and biosimilar products and calculated a dose-weighted mean of the Medicare payment limit. We classified drugs with generics or biosimilars by October 2020 as those facing competition and all others as single-source brand drugs.

### Statistical Analysis

Statistical analysis was performed from June to December 2022 using Stata, version 17.0 (StataCorp LP). Increases in spending for Part B drugs may reflect changes in price, quantity, and/or the composition of products. To better understand these dynamics, we quantified the concentration of spending and identified the number and types of top-spending Part B drugs over time. We then compared drug use, in terms of both beneficiaries and dosage units, across products in each quartile of the Medicare payment limit. To account for differences in dosage units across products, we replicated this analysis using mean spending per beneficiary.

We then decomposed the increase in Part B spending from 2016 to 2020 into changes associated with price and use for 3 subsamples of drugs based on the presence or absence of competition and the first year of administration. Existing drugs were those administered to Medicare beneficiaries at the start of the sample and appeared in the panel as of 2016; new drugs were those that launched after 2016 and entered the panel between 2017 and 2020.

We also computed 2 indices that reveal price and use dynamics over time among the subsample of existing drugs. For the first index, we calculated the cost of purchasing a fixed set of drugs at current-year prices and divided it by the cost of that same drug set in 2016. By holding the drug products and quantities constant, this index isolated the association of the Medicare payment limit with changes in total spending. The second index measured the association of changes in quantity with spending by fixing the price of each product at its Medicare payment limit in 2016. We calculated the cost of each annual drug set and divided by the cost of the 2016 drug set. We multiplied the results of both indices by 100, so relative increases (or decreases) in the cost of a set of drugs are indicated by index values over (or under) 100.

## Results

**Figure 1.**
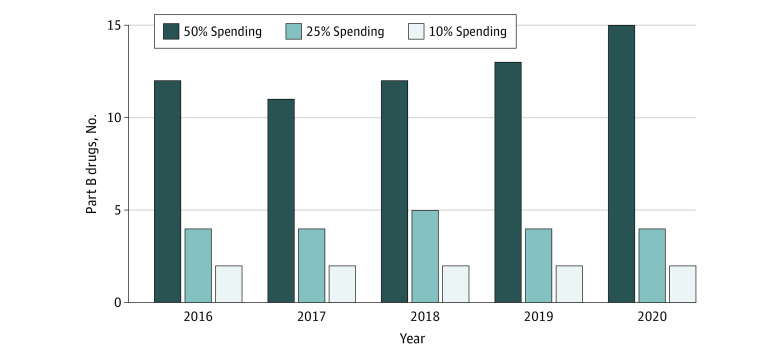
Concentration of Medicare Part B Drug Spending, 2016-2020

The study included 535 unique Part B drug products. Among the drugs in our sample, annual Part B drug spending increased from $24.5 billion in 2016 to $39.5 billion in 2020. Spending was highly concentrated, with half of all expenditures in each year associated with 15 or fewer products (Figure 1). The size of this top-spending group increased over time, suggesting greater dispersion, but there was a high degree of consistency among the products represented (Table). During our study period, 7 drugs comprised the top 25% of spending, and all were biologics. In 2020, the immunotherapy drug pembrolizumab overtook the retinal disease treatment aflibercept as the top-spending Part B drug. These 2 drugs accounted for nearly 15% of annual Part B drug spending over the study period.

**Table.** Characteristics of Top Spending Medicare Part B Drugs, 2016-2020^a^

Brand name	Generic name	Therapeutic area	2020 spending (billions), $	Top 25% of spending, by year				
				2016	2017	2018	2019	2020
Keytruda	Pembrolizumab	Cancer	3.50	No^b^	No	Yes	Yes	Yes
Eylea	Aflibercept	Eye conditions	3.01	Yes	Yes	Yes	Yes	Yes
Prolia or Xgeva	Denosumab	Osteoporosis	1.63	No	No	Yes	No	Yes
Opdivo	Nivolumab	Cancer	1.59	No^b^	Yes	Yes	Yes	Yes
Rituxan	Rituximab	Cancer, rheumatoid arthritis	1.30	Yes	Yes	Yes	Yes	No
Neulasta	Pegfilgrastim	Cancer adverse effects	0.90	Yes	Yes	No	No	No
Remicade	Infliximab	Rheumatoid arthritis	0.66	Yes	No	No	No	No

^a^ This table lists drugs that comprised the top 25% of total Part B spending in each year. For drugs with a biosimilar, spending reflects only the reference biologic. Data reflect fee-for-service Medicare Part B claims for separately payable drugs with a valid Medicare payment limit and Healthcare Common Procedure Coding System code throughout the 2016-2020 period. Excludes Part B claims with Not Otherwise Classified codes and submissions from clinicians outside of the Outpatient Prospective Payment System.

^b^ Not yet in the Part B drug panel.

**Figure 2.**
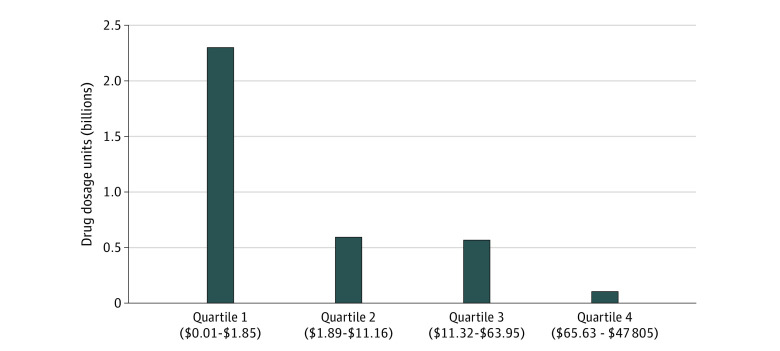
Concentration of Medicare Part B Drug Dosage Units by Quartiles of the Medicare Payment Limit, 2020

Part B drug use, however, was concentrated among lower-priced products (Figure 2). In 2020, over half the 3.5 billion dosage units of Part B drugs were for products in the bottom quartile of the Medicare payment limit (≤$1.85). Drugs in the top quartile of the Medicare payment limit that cost $66 or more per unit accounted for less than 3% of all administrations. Because drug treatment episodes may vary in terms of dosage units, we also examined the number of beneficiaries using these products. The results are similar, with a greater concentration of beneficiaries using the drugs in the lower half of the Medicare payment limit distribution (eFigure 2 in Supplement 1). Meanwhile, Part B drug spending was concentrated among higher-priced products, whether measured as total dollars or as a per-beneficiary mean. Drugs with an above-median Medicare payment limit accounted for nearly 90% of total dollars spent on Part B drugs (eFigure 3 in Supplement 1). Similarly, mean Medicare Part B spending per beneficiary was highest for drugs in the top quartile of the payment limit (eFigure 4 in Supplement 1).

**Figure 3.**
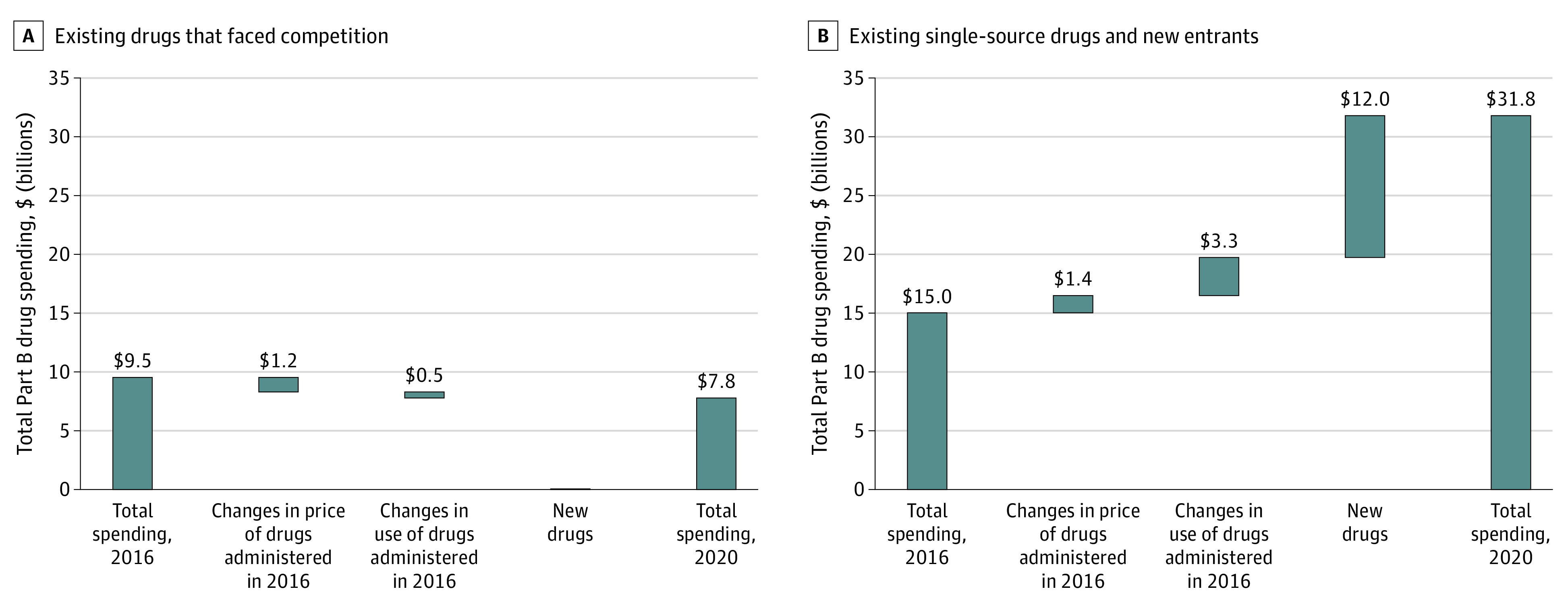
Decomposing Changes in Medicare Part B Drug Spending, 2016-2020 Sums or differences may not match exactly due to rounding.

Although changes in price and use for existing single-source drugs were associated with Part B spending increases from 2016 to 2020, the entry of new products had a relatively greater association with the increase in spending (Figure 3). In 2020, the Medicare program and its beneficiaries spent $12 billion on nonbiosimilar products that entered the market after 2016. In contrast, spending decreased among existing drugs that faced competition by the end of the sample period. Combined changes in price—and to a lesser extent in use—reduced spending by nearly $2 billion. Three-quarters of these drugs already had generics or biosimilars, although the newly competitive group comprised 80% of spending at the start of our sample period. From 2016 to 2020, total spending decreased for both subgroups of drugs facing competition. In all, 80% of Part B spending increases from 2016 to 2020 were associated with the introduction of new drugs, with much of the remainder stemming from changes in the use of existing drugs that lack competitor products.

**Figure 4.**
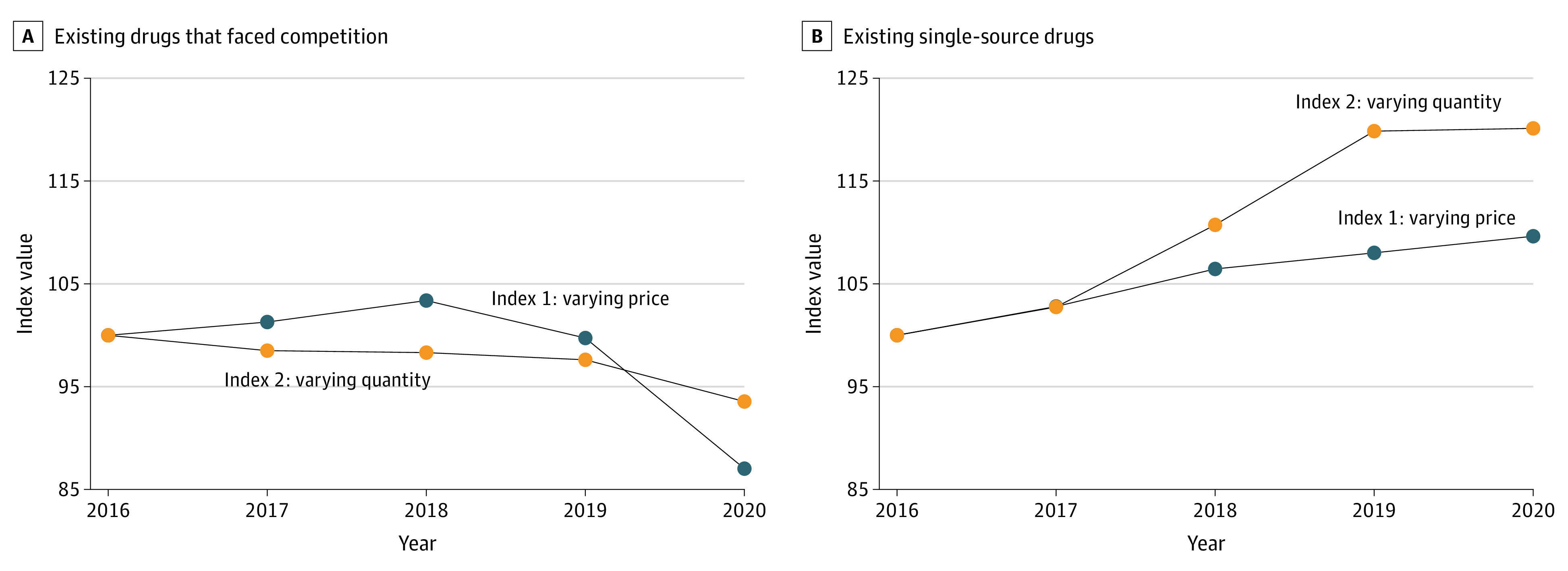
Medicare Part B Drug Index Analysis, 2016-2020

Index analyses confirmed that, among existing single-source Part B drugs, a relatively larger share of spending increases through 2020 could be associated with changes in dosage units than with changes in price (Figure 4). Relative to the baseline level of 100, the index that varied dosage units peaked 10 percentage points higher than the index that varied the payment limit. The gap between the 2 indices also widened over time, with the greatest change occurring between 2018 and 2019, when unit prices increased slightly, and the number of dosage units had a large increase. For existing drugs that faced competition, much of the decreases in price occurred between 2019 and 2020; this finding is consistent with launch patterns for biosimilar drugs.

## Discussion

Our analysis of Medicare Part B drug spending shows that relatively few biologic drugs comprised most expenditures. Related work has shown that most biologic drugs lack biosimilar competitors in both the current market and the pipeline.^2^ High-spending, single-source drugs that have been on the market for a number of years will likely be subject to price negotiations under the Inflation Reduction Act of 2022. Negotiations may curb future increases in the Medicare payment limit.

We also found that, compared with existing products, drugs more recently brought to market were associated with Part B spending increases in recent years. These findings complement prior work that found nearly all increases in Part B drug spending over the last decade were associated with biologics.^2^ Although CMS cannot negotiate prices of newly marketed drugs, prices are subject to the inflation rebate. The association of these provisions with spending is still unclear, however, given that just 20 Part B drugs will be included in the first round of inflation rebates.^5^ We also found that the price of single-source drugs accounted for a relatively small share of the overall increase in spending, which suggests that the association of the rebate previsions with spending may be muted.

In addition, index analyses revealed that the relative association of price and use factors with spending varied over time, with the latter accounting for a larger share of increased spending among existing single-source drugs. Given that enrollment in FFS Medicare Part B decreased 4% over this period, our results suggest that per-enrollee use increased more rapidly than overall use.^6^

These results have implications for the Part B reimbursement policy, which is unique in its “buy and bill” model (eFigure 5 in Supplement 1). Under this system, clinicians and HOPDs purchase drugs in advance and bill Medicare after administration. Recent work has suggested that this reimbursement model rewards high launch prices and the use of high-cost products.^7,8,9^ Our analysis suggests that policies targeting the top-selling drugs have the greatest potential to curb spending, while those targeting price increases will have smaller effects.

### Limitations

This study had several limitations. Most notably, drug spending and use for Medicare Advantage, which constitutes more than 40% of total Medicare enrollment in 2020, was not captured by these data.^10^ Use of Part B drugs may differ between FFS and Medicare Advantage enrollees, either because of different health needs or because of utilization management tools in Medicare Advantage plans. To the extent that drug use or payments differ between the 2 populations, our analysis may not be representative of the Medicare program as a whole. To a lesser degree, these data were also limited in their focus on clinicians paid under the Medicare Physician Fee Schedule and the Outpatient Prospective Payment System. These data also excluded drugs administered in HOPDs that are packaged for payment along with other services, which accounted for 5% of total spending on Part B drugs.^1^ These annualized measures also obscured the fact that drugs entering the market are not fully represented in their first year, although much of our analysis distinguishes between new and existing drugs. Measures of aggregate beneficiary use should be interpreted as an upper bound because we measured drug use at the drug-year level, and beneficiaries often use more than 1 drug. Information for a given drug-year observation may also be missing due to changes in reporting requirements or product discontinuation. Per CMS disclosure guidelines, the data set excluded drug-year observations with fewer than 11 Part B FFS claims. We observed 394 drugs (74% of the 535 unique identified products) for all 4 years. Our analysis period also covered the onset of the COVID-19 public health emergency, which was associated at least in part with slower increased spending patterns in 2020 relative to prior years.^1^

## Conclusions

In this cross-sectional study of Medicare Part B drugs, we found that program and beneficiary spending was concentrated among a small number of drug products from 2016 to 2020, and many top-spending drugs were biologics. Although increases in use and prices of existing products played a role, market entry of new products was the primary factor associated with increases in Part B drug spending during this period. The findings suggest that policies targeting top-selling drugs may have greater potential to curb Part B drug spending than those targeting price increases.

## Notes

Supplement 1. **eFigure 1.** Construction of Analysis Sample **eFigure 2.** Concentration of Medicare Beneficiaries Using Part B Drugs by Quartiles of the Medicare Payment Limit, 2020 **eFigure 3.** Concentration of Medicare Part B Spending by Quartiles of the Medicare Payment Limit, 2020 **eFigure 4.** Average Medicare Part B Spending per Beneficiary by Quartiles of the Medicare Payment Limit, 2020 **eFigure 5.** Medicare Part B Drug Reimbursement Model

Supplement 2.
Data Sharing Statement
