# Supplementary material for: Spending on and Use of Clinician-Administered Drugs in Medicare
Source: JAMA Health Forum. 2023 Sep 8;4(9):e232941. doi: 10.1001/jamahealthforum.2023.2941 (PMC10492179; doi:10.1001/jamahealthforum.2023.2941)
Supplement: Supplement 1. — eFigure 1. Construction of Analysis Sample eFigure 2. Concentration of Medicare Beneficiaries Using Part B Drugs by Quartiles of the Medicare Payment Limit, 2020 eFigure 3. Concentration of Medicare Part B Spending by Quartiles of the Medicare Payment Limit, 2020 eFigure 4. Average Medicare Part B Spending per Beneficiary by Quartiles of the Medicare Payment Limit, 2020 eFigure 5. Medicare Part B Drug Reimbursement Model [file jamahealthforum-e232941-s001.pdf]

Supplementary Online Content

Hyland MF, Sachs RM, Robillard L, Hayford TB, Bai G. Spending on and use of clinician-administered drugs in Medicare. *JAMA Health Forum.* Published online September 8, 2023. doi:10.1001/jamahealthforum.2023.2941

eFigure 1. Construction of Analysis Sample
eFigure 2. Concentration of Medicare Beneficiaries Using Part B Drugs by Quartiles of the Medicare Payment Limit, 2020
eFigure 3. Concentration of Medicare Part B Spending by Quartiles of the Medicare Payment Limit, 2020
eFigure 4. Average Medicare Part B Spending per Beneficiary by Quartiles of the Medicare Payment Limit, 2020
eFigure 5. Medicare Part B Drug Reimbursement Model

This supplementary material has been provided by the authors to give readers additional information about their work.

© 2023 Hyland MF et al. *JAMA Health Forum.*

eFigure 1. Construction of Analysis Sample[a]

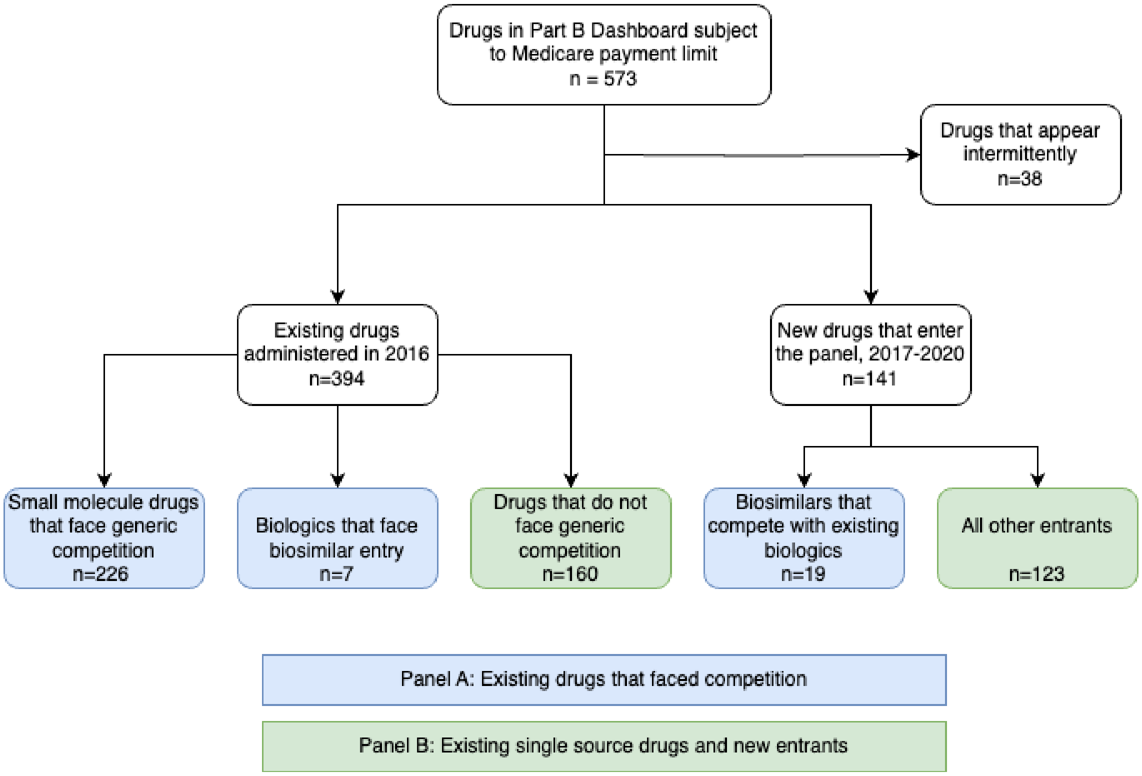

[a] This figure shows the sample creation process, beginning with the drugs paid under the Medicare Payment limit (ASP) in the Medicare Part B Spending by Drug data, 2016-2020. In the final row, blue cells indicate that the drugs are included in Panel A of Figures 3 and 4; green cells indicate that the drugs are included in Panel B.

© 2023 Hyland MF et al. *JAMA Health Forum*.

eFigure 2. Concentration of Medicare Beneficiaries Using Part B Drugs by Quartiles of the Medicare Payment Limit, 2020[a]

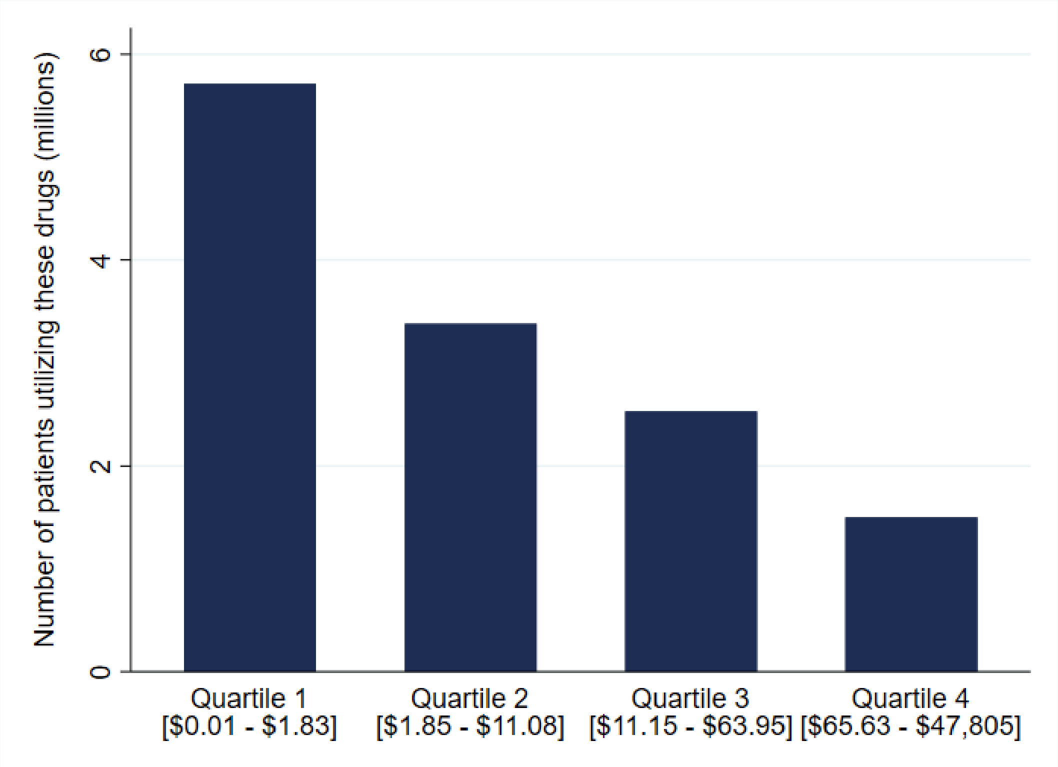

[a] This figure shows the number of beneficiaries using the drugs in a given quartile of the Medicare payment limit in 2020. Beneficiary counts are censored due to small cell size for 21 drugs, so there are 129 drugs in quartiles 1 and 3 and 128 in quartiles 2 and 4. Dollar values on the x-axis show the Medicare payment limit range covered by that quartile. Data reflect fee-for-service Medicare Part B claims for separately payable drugs with a valid Medicare payment limit and Healthcare Common Procedure Coding System code throughout the 2016-2020 period. Excludes Part B claims with Not Otherwise Classified Codes and submissions from clinicians outside of the Outpatient Prospective Payment System.

© 2023 Hyland MF et al. *JAMA Health Forum*.

eFigure 3. Concentration of Medicare Part B Spending by Quartiles of the Medicare Payment Limit, 2020[a]

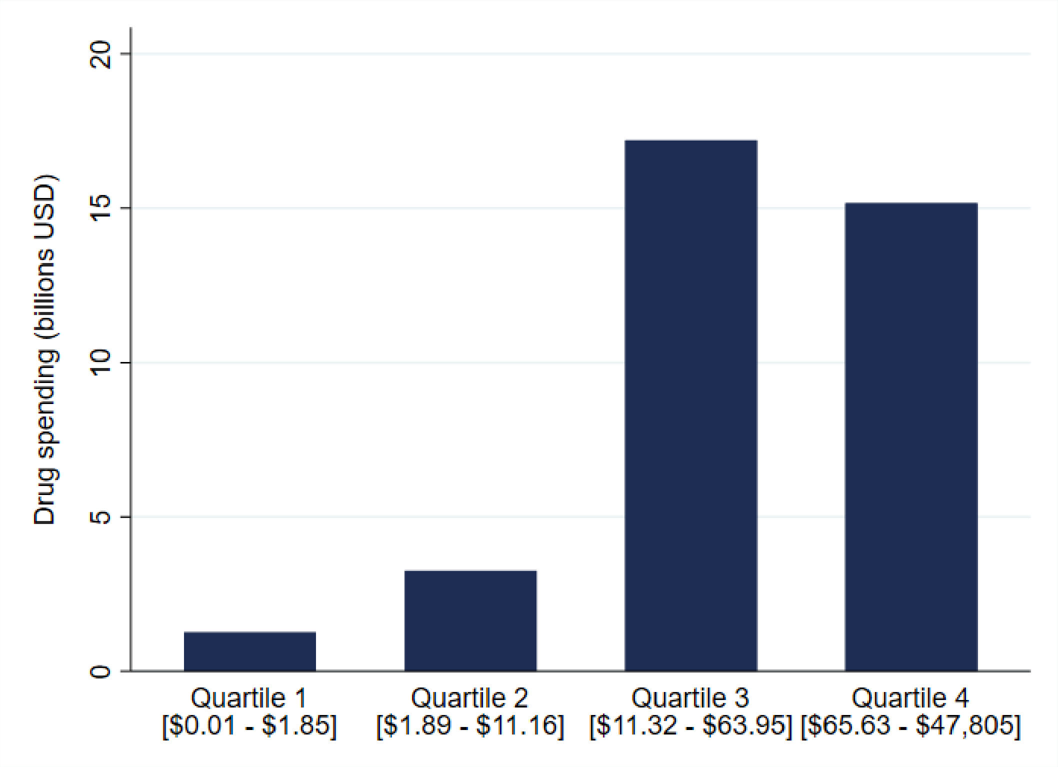

[a] This figure shows total spending for drugs in a given quartile of the Medicare payment limit in 2020. There are 134 drugs in quartiles 1 through 3 and 133 in quartile 4. Dollar values on the x-axis show the Medicare payment limit range covered by that quartile. Data reflect fee-for-service Medicare Part B claims for separately payable drugs with a valid Medicare payment limit and Healthcare Common Procedure Coding System code throughout the 2016-2020 period. Excludes Part B claims with Not Otherwise Classified Codes and submissions from clinicians outside of the Outpatient Prospective Payment System.

© 2023 Hyland MF et al. *JAMA Health Forum*.

eFigure 4. Average Medicare Part B Spending per Beneficiary by Quartiles of the Medicare Payment Limit, 2020[a]

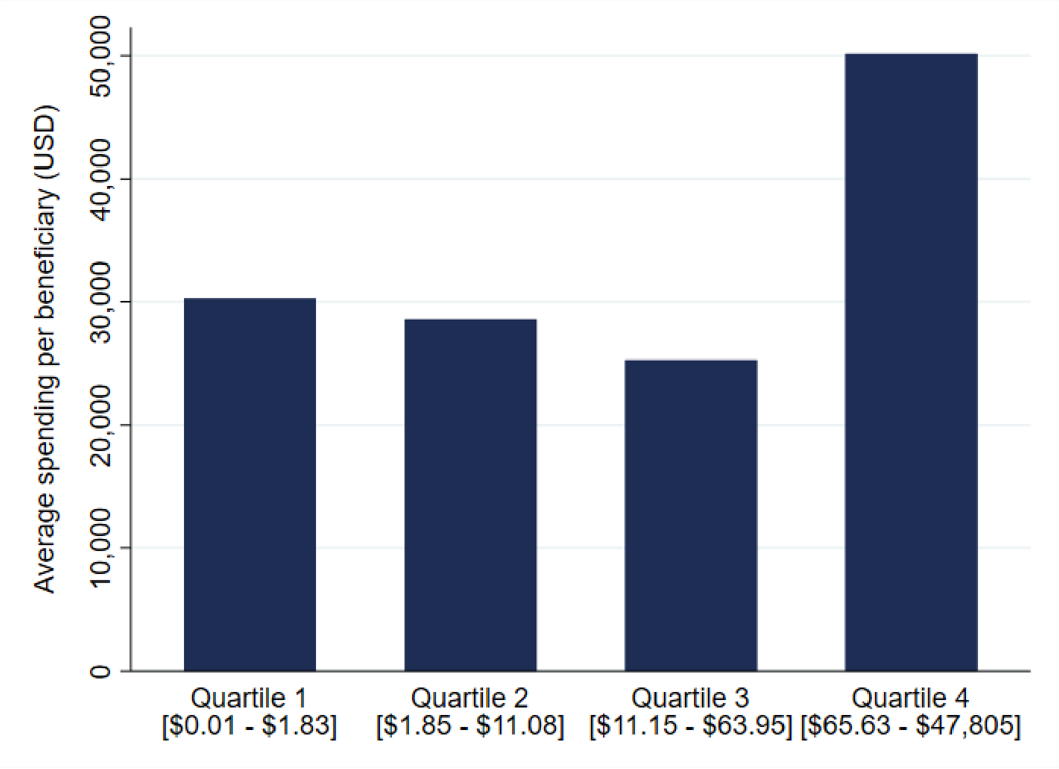

[a] This figure shows average spending per beneficiary using the drugs in a given quartile of the Medicare payment limit in 2020. Beneficiary counts are censored due to small cell size for 21 drugs, so there are 129 drugs in quartiles 1 and 3 and 128 in quartiles 2 and 4. Dollar values on the x-axis show the Medicare payment limit range covered by that quartile. Data reflect fee-for-service Medicare Part B claims for separately payable drugs with a valid Medicare payment limit and Healthcare Common Procedure Coding System code throughout the 2016-2020 period. Excludes Part B claims with Not Otherwise Classified Codes and submissions from clinicians outside of the Outpatient Prospective Payment System.

© 2023 Hyland MF et al. *JAMA Health Forum*.

eFigure 5. Medicare Part B Drug Reimbursement Model[a]

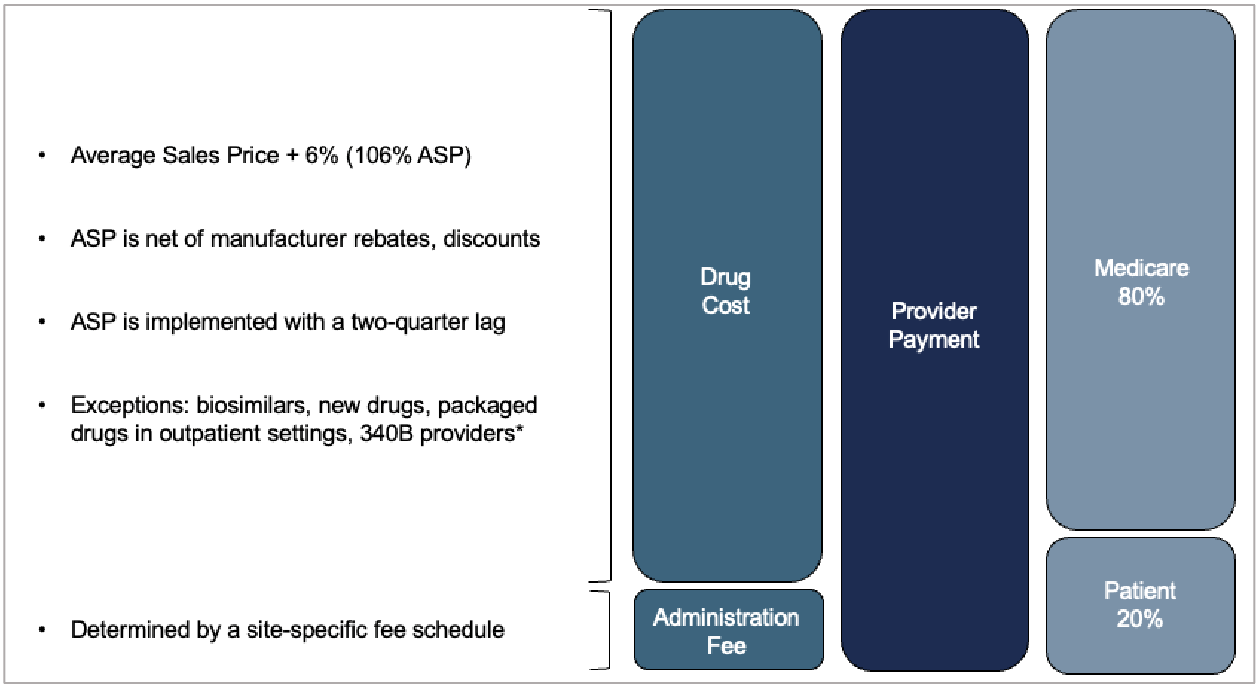

[a] As of calendar year 2018 Outpatient Prospective Payment System final rule, 340B-acquired drugs were reimbursed at ASP-22.5%. However, CMS has announced it will revert to paying 340B providers ASP+6% for 340B drugs.

© 2023 Hyland MF et al. *JAMA Health Forum.*
