# Supplementary material for: Spending on and Use of Clinician-Administered Drugs in Medicare
Source: JAMA Health Forum. 2023 Sep 8;4(9):e232941. doi: 10.1001/jamahealthforum.2023.2941 (PMC10492179; doi:10.1001/jamahealthforum.2023.2941)
Supplement: Supplement 2. — Data Sharing Statement [file jamahealthforum-e232941-s002.pdf]

Data Sharing Statement

Hyland. Spending on and Use of Clinician-Administered Drugs in Medicare. *JAMA Health Forum*. Published September 08, 2023. doi:10.1001/jamahealthforum.2023.2941

Data
Data available: No

Additional Information
Explanation for why data not available: The dataset used in this study is publicly available.
